# Molecular profile of atypical Leydig cell tumours

**DOI:** 10.1111/his.70018

**Published:** 2025-10-09

**Authors:** Muhammad F.K. Choudhry, Diogo Caires, Yaser Gamallat, Asli Yilmaz, Fadi Brimo, Bob Argiropoulos, Tarek A. Bismar

**Affiliations:** ^1^ Department of Pathology & Laboratory Medicine University of Calgary Cumming School of Medicine & Alberta Precision Labs Calgary Alberta Canada; ^2^ Department of Clinical Laboratory Medicine McGill University Health Centre Montreal Quebec Canada

## Abstract

**Aims:**

To investigate histological and copy number variations (CNVs) in Leydig cell tumours (LCTs) of the testis. Although usually benign, a small minority of cases can be associated with a poor prognosis and metastasis.

**Methods:**

We performed whole copy number analysis to compare the genomic profile of atypical (defined by the presence of any atypical features) versus benign LCTs. Our sample consisted of one malignant (with biopsy‐proven metastasis), five atypical and five benign cases.

**Results:**

We found increased genomic instability in the malignant tumour and within two out of five (40%) atypical cases. One benign case revealed a likely pathogenic mutation in the neurofibromatosis type 2 gene, but all benign cases lacked genomic instability. Apart from the malignant case (which had metastatic spread to the scrotal skin), all remaining atypical cases did not reveal evidence of recurrence or metastatic spread.

**Conclusion:**

CNVs by themselves are not sufficient to discriminate between cases that are benign versus those with malignant potential, without the use of histomorphological parameters. Genomic instability was only detected in the malignant and atypical cases, and not in any of the benign tumours. Thus, genomic instability may represent an early step in malignant progression. The presence of metastasis remains the only malignant criterion for LCTs.

## Graphical abstract

Copy number variations by themselves are not sufficient to discriminate between benign and potential malignant Leydig cell tumours (LCTs). Genomic instability was only detected in malignant and atypical cases, and not in any benign tumours. Currently, the presence of metastasis remains the only malignant criterion for LCTs.

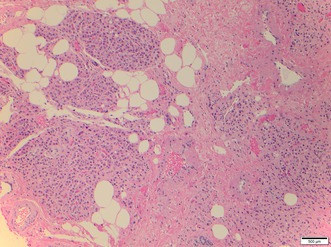

**Abbreviations**
AFP: alpha fetoprotein
b‐HCG: human chorionic gonadotropin beta subunit
CDK4: cyclin‐dependent kinase 4
CNA: copy number alteration
CNV: copy number variation
FFPE: formalin‐fixed paraffin‐embedded
GA: genomic alteration
GCNIS: germ cell neoplasia in situ
H&E: haematoxylin and eosin
IHC: immunohistochemistry
LCT: Leydig cell tumour
LDH: lactate dehydrogenase
LeSS: Leydig cell tumour Scaled Score
MDM2: mouse double minute 2
NGS: next‐generation sequencing
SCST: sex cord‐stromal tumour
SNP: single nucleotide polymorphism

## Introduction

Leydig cell tumours (LCTs) are the most common sex cord‐stromal tumours (SCSTs) of the testis, although they represent only 1%–3% of all testicular tumours.^1^, ^2^ The majority of LCTs display a benign behaviour, with orchiectomy being curative for most patients. However, in a minority of cases, they can be associated with a poor prognosis and metastasis.^3^, ^4^ Some effort has been placed in determining the characteristics that allow for stratification of LCTs based on their risk of progression. One of the first studies to evaluate the impact of adverse pathological parameters was that conducted by Kim *et al*. in which six key parameters of malignancy were proposed, including >3 mitoses/10 HPFs, nuclear atypia, vascular invasion, infiltrative borders, necrosis and size >50 mm. Their study contained a total of five malignant LCTs, with proven metastatic spread and death to the patient. Malignant cases, when compared to 14 benign LCTs (showing lack of metastatic spread), revealed a larger size and a higher frequency of infiltrative borders and spread beyond the testis, blood vessel or lymphatic invasion, a greater degree of nuclear atypia, the presence of necrosis and a higher mitotic rate.^5^ The authors did not suggest a scoring method, although certain cut‐offs for adverse parameters were suggested, as discussed. Since this seminal project, few groups have tried to characterise the prognosis of LCTs based on clinicopathologic features. A study published by Cheville *et al*. corroborated most of the findings by Kim's group. In addition, they reported a higher rate of DA aneuploidy and MIB‐1 activity in metastatic tumours.^6^ In 2010, a group by Heer *et al*. analysed the outcomes of 29 LCTs and found that the previously described adverse prognostic parameters were only infrequently observed in their cohort. The group hypothesised that the rate of metastatic progression for these tumours overall is likely below the previously reported rate of 10%. Unlike the previous group, they did not have any malignant cases for comparison.^7^ More recently, an Italian group, led by Colecchia, developed a new scoring system for LCTs called the Leydig cell tumour Scaled Score (LeSS). The scoring involves five adverse factors (mitoses, size, necrosis, infiltrative pattern and vascular invasion). The presence of necrosis, infiltrative pattern and vascular invasion all receive a score of 1, whereas mitoses and size can each receive a maximum of 2 points based on their proposed cut‐offs of 1–3/10 HPF and ≥4/10 HPF for mitoses and 15 to ≤25 mm and >25 mm for size, respectively. Their study showed that a LeSS ≥4 correctly identified 14/14 malignant cases whereas a score <4 correctly identified 37/37 benign ones.^8^ To note, the cut‐offs chosen for mitotic rate and tumour size do deviate from previously suggested values. In addition, nuclear atypia was not included in their scoring system.

Despite advances in the development of histomorphological criteria for predicting the behaviour of LCTs, there is still a paucity of data in the literature with regard to the molecular differences between benign and aggressive cases. Specifically, there is minimal to no literature available on genomic alterations (GAs) affecting LCTs that possess some atypical/adverse features, but that are not yet malignant (i.e. negative for metastatic spread). This is an important area of study as it could provide another tool for the detection of potentially aggressive tumours early in their development so that they can be treated before they spread outside of the testis. In the present study, we attempt to shed further light on the genomic characteristics of such LCTs by performing whole copy number profiling of 11 testicular LCTs. Five of these cases show no adverse prognostic factors, as originally proposed by Kim *et al*. and are deemed ‘benign’. Five other cases reveal the presence of at least one adverse factor (e.g. nuclear atypia) and are designated as ‘atypical’. Lastly, 1 case shows metastatic spread and is termed ‘malignant’.

## Methods

### Collection of Clinical Information

Patients' electronic medical records were retrospectively accessed by using Citrix's software and Alberta Netcare. All patients were followed from the day of diagnosis until their last follow‐up or their death. Examples of follow‐up included urological consultations, scrotal imaging by ultrasound, magnetic resonance imaging or computed tomography (CT) and assessment of tumour biomarkers including alpha fetoprotein (AFP), human chorionic gonadotropin beta subunit (b‐HCG) and lactate dehydrogenase (LDH). Clinical parameters included age at the time of diagnosis, type of treatment, presence of local or distant recurrence and death at last follow‐up. Laboratory parameters at the time of diagnosis included AFP, LDH and b‐HCG.

### Collection of Pathologic Parameters

Histopathologic review of glass slides (haematoxylin and eosin [H&E] stains) was conducted by a single observer (A.Y.). Tumour size and presence of infiltrative margins were extracted from gross reports, accessed on the Millennium software database. Pathologic parameters included size, infiltrative margins, necrosis, vascular invasion, mitoses (per 10 high power fields, /10 HPF), nuclear atypia and presence of germ cell neoplasia in situ (GCNIS). LeSS scores were calculated for each tumour, according to the scoring method presented in the original publication by Collechia *et al*.

### Assessment of Copy Number Variations (CNVs)

#### OncoScan single nucleotide polymorphism array patient cohort

We assessed 11 cases from formalin‐fixed paraffin‐embedded (FFPE) tissues with the OncoScan single nucleotide polymorphism (SNP) platform. Tissue slides were procured and stained with H&E. The best tumour area was selected from each slide by the interpreting pathologist. Three unstained slides were cut for each block and circled areas were microdissected. Tumour tissue was collected in Eppendorf tubes. Genomic DNA was extracted using the Qiagen FFPE DNA extraction kit and subsequently quantified. DNA samples were sent to Yale University for OncoScan SNP microarray profiling. Ethics approval was obtained from the Research Ethics Committee.

#### SNP microarray data generation and copy number alteration calling

SNP microarrays were performed with 200 ng of DNA on Affymetrix OncoScan FFPE Express 3.0 arrays. The SNP genotypes were extracted from the OncoScan OSCHP files. Genomic copy number alterations (CNAs) for each patient were identified by overlapping copy number (CN) segments; annotation was done manually.

#### Analysis of affymetrix OncoScan

OncoScan 3.0 array data was analysed from the OSCHP files and generated by the OncoScan Console. The OncoScan 3.0 Software was used to call CNAs using the SNP‐FASST2 algorithm with default parameters. The minimum number of probes per segment was changed from 3 to 20. Gene level CNAs for each patient were identified by overlapping CN segments, with RefGene annotation. To account for technical noise, CNVs used GISTIC2.0 (v2.0.22) to study the gene level CNVs in our sample set. As input to GISTIC2.0, a profile for each sample was created that segmented each chromosome into regions with neutral, CN loss and CN gain events. The average copy number intensity for each segment was obtained from the SNP array analysis.

## Results

### Population Characteristics

The clinical features of the 11 cases selected for this study are listed in Table 1. Age varied from 26 to 72 years. Tumour markers were within normal limits for all cases, with minor variations as shown. Laterality was equally distributed, with five cases located on the right and five on the left testicle. All patients received an orchiectomy as part of their treatment. Length of follow‐up varied significantly between each case from no follow‐up (patient discharged from care after surgery) to 108 months at its longest. Only one case (case #6) showed recurrence, with tumoural lesions being identified on the ipsilateral scrotal skin approximately 4 years after surgery. Three patients died at last follow‐up, all of them unrelated to their LCT. All three patients had concomitant metastatic malignancies (cases #4, #8 and #10) and died as a result of their metastatic disease burden.

**Table 1:** Clinical and laboratory parameters for all 11 Leydig cell tumours

Case	Age (years)	AFP (ug/L)	LDH (U/L)	bHCG (IU/L)	Side	Treatment	F/U (months)	Recurrence	Death at last F/U	Other
1	25	1.4	125	<1	Right	Orchiectomy	5	No	No	
2	67	2.7	205	<1	Left	Orchiectomy	45	No	No	
3	45	5.5	190	<1	Right	Orchiectomy	3	No	No	
4	66	2.5	257	<1	Left	Orchiectomy	31	No	Yes	Liposarccoma
5	53	5.1	n/a	<1	Left	Orchiectomy	108	No	No	
6	71	n/a	n/a	n/a	Right	Orchiectomy	72	Yes	No	
7	59	n/a	n/a	n/a	Left	Orchiectomy	96	No	No	
8	48	n/a	n/a	n/a	Right	Orchiectomy	60	No	Yes	Lung adenocarcinoma
9	46	3.2	n/a	<1	Left	Orchiectomy	0	No	No	
10	72	3.5	207	<1	Right	Orchiectomy	0	No	Yes	Merkel cell carcinoma
11	n/a	n/a	n/a	n/a	n/a	Orchiectomy	12	No	No	

AFP, alpha fetoprotein; bHCG, human chorionic gonadotropic; beta subunit; F/U, follow‐up; LDH, lactate dehydrogenase; n/a, not available.

### Pathologic Findings

**Figure 1:**
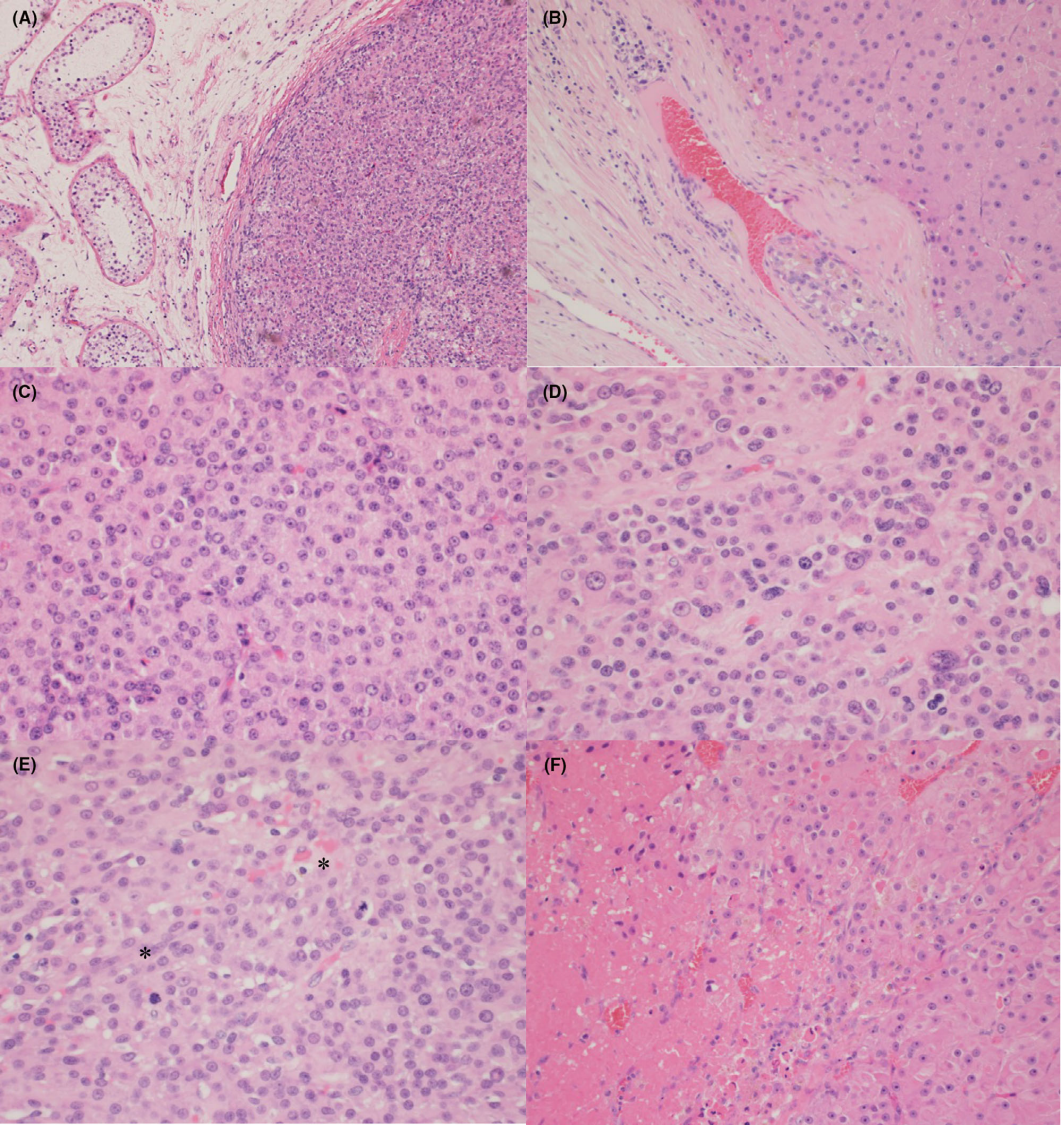
Representative histological images from our cohort: (**A**) Benign Leydig cell tumour (LCT) with well‐circumscribed borders in contrast with (**B**) an atypical LCT with an irregular tumour‐stromal interface. (**C**) Benign LCT with monotonous cytology compared with (**D**) an atypical LCT with focal moderate nuclear atypia. (**E**) Atypical LCT with increased mitotic figures; *Two mitoses are identified in this one HPF. (**F**) Atypical LCT with abrupt transition to an area of necrosis.

**Figure 2:**
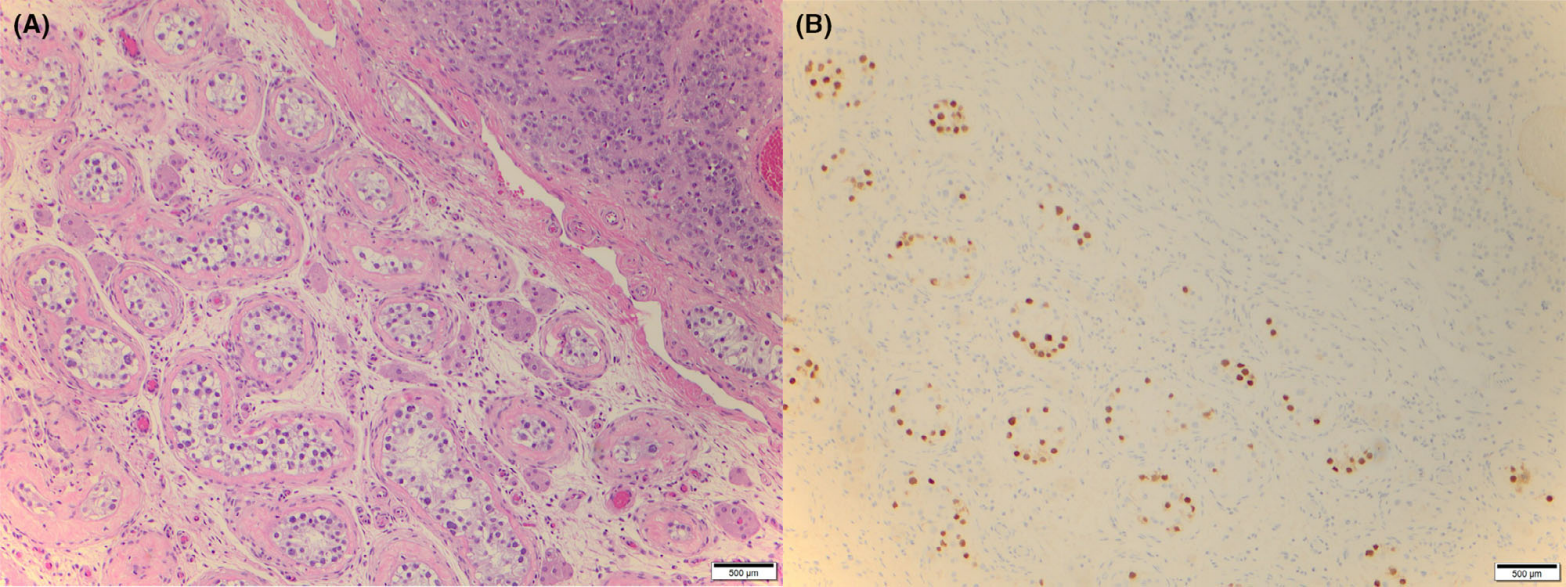
Atypical LCT patient demonstrating the presence of GCNIS. (**A**) H&E stain of GCNIS adjacent to Leydig cell tumour. (**B**) IHC staining of OCT4 (10× magnification).

Histological parameters assessed for all 11 cases are displayed in Table 2. In total, five cases were categorized as benign, five atypical and one malignant, as previously defined. Tumour size varied from 0.7 cm (case #4, atypical) to 5.5 cm (case #6, malignant). Only one case revealed an infiltrative pattern of growth (case #2, atypical; Figure 1B), while two cases showed the presence of necrosis and vascular invasion (case #2, atypical and case #6, malignant; Figure 1B,F). Mitoses ranged from 0 (four cases) to 3–5/10 HPF (case #5, atypical; mitotic figures with atypical forms were also identified). All atypical and the one malignant case revealed the presence of nuclear atypia (Figure 1D), which was absent in all benign cases. GCNIS was incidentally noted in only one tumour (case #1, atypical; Figure 2). LeSS scores varied widely from 0 (case #4, atypical) to 6 (case #6, malignant). Lastly, genomic instability was identified in two atypical tumours (cases #1 and #2) and in the malignant case (#6). Genomic analysis is discussed in the next section.

**Table 2:** Pathologic and genomic parameters for all 11 Leydig cell tumours

Case	Greatest dimension (cm)	Infiltrative margins	Necrosis	Vascular invasion	Mitoses (/10 HPF)	Nuclear atypia	GCNIS	Type	LeSS	Genomic instability
1	1.1	No	No	No	3	Yes	Yes	Atypical	1	Yes
2	2.5	Yes	Yes	Yes	3	Yes	No	Atypical	5	Yes
3	2.0	No	No	No	0	Yes	No	Atypical	1	No
4	0.7	No	No	No	0	Yes	No	Atypical	0	No
5	3.7	No	No	No	3–5*	Yes	No	Atypical	4	No
6	5.5	No	Yes	Yes	5	Yes	No	Malignant	6	Yes
7	2.0	No	No	No	0	No	No	Benign	1	No
8	3.2	No	No	No	<3	No	No	Benign	3	No
9	2.4	No	No	No	1	No	No	Benign	2	No
10	2.2	No	No	No	0	No	No	Benign	1	No
11	2.5	No	No	No	1	No	n/a	Benign	2	No

GCNIS, germ cell neoplasia in‐situ; HPF, high‐power field; LeSS, Leydig cell tumour Scaled Score.

* Rare atypical mitotic figures were also identified.

### Copy‐Number Alteration (CNA) Analysis

**Figure 3:**
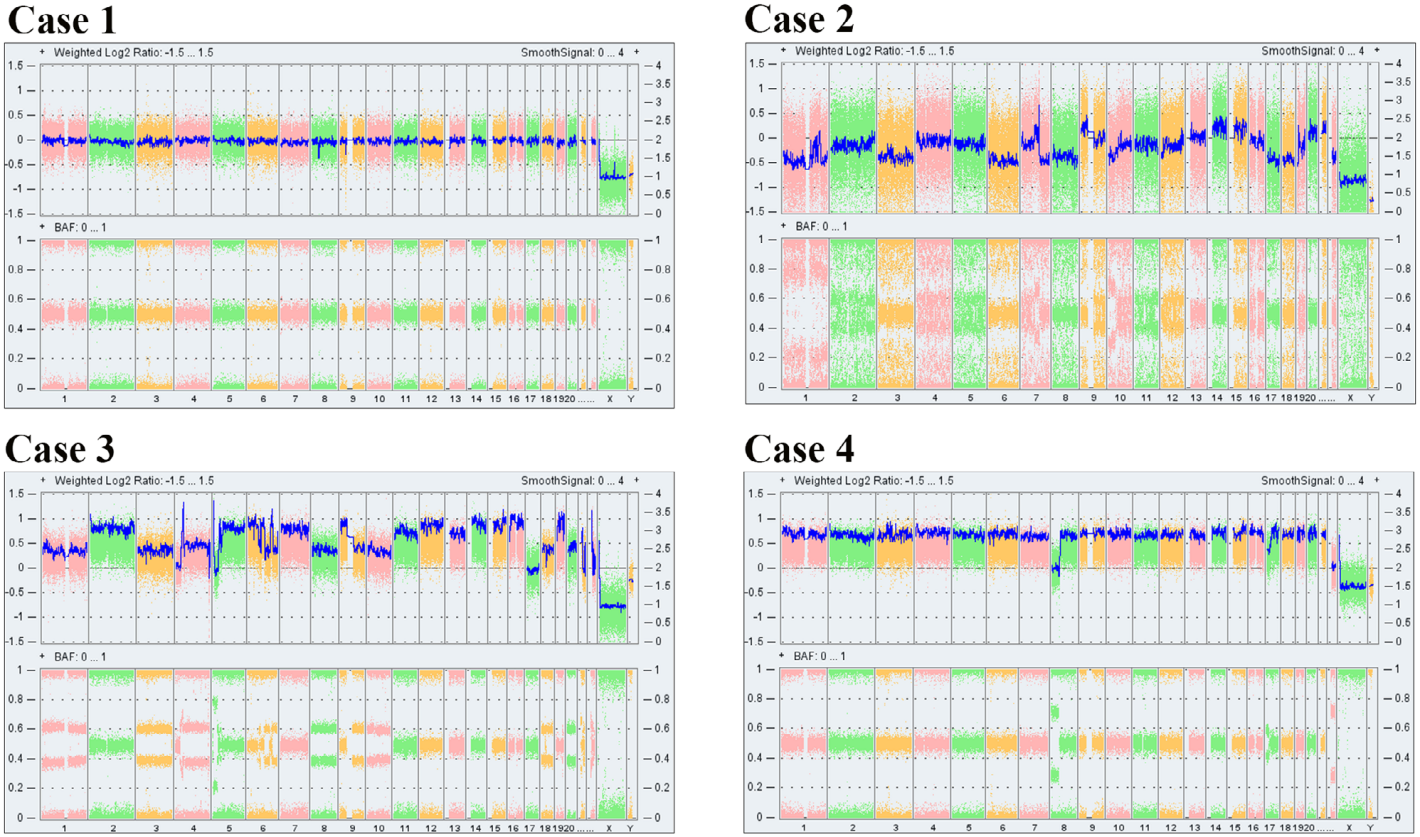
Whole genome profiles of tumour DNA analysed by OncoScan from four cases showing the copy number status (top panel – log2 ratio) and zygosity (B‐allele frequency) (bottom panel). Case 1 shows a genomic profile without gross aberrations. A 172 kb mosaic copy number loss within chromosome 22q12.2 overlapping the NF2 gene was detected. Cases 2, 3 and 4 exhibit evidence of genomic instability with evidence of multiple partial or complete monosomies (case 1), multiple partial or complete trisomies (case 2) and ploidy changes with regions showing copy‐neutral loss of heterozygosity (case 3). This specimen showed increased copy number (4n) of the majority of the genome with a balanced allele distribution. According to the BAF, there are 3 copies of chromosome 8p and 22 (case 4).

CNA analysis was performed for all 11 LCTs using the OncoScan™ CNV Assay by Thermo Fisher, which performs whole genome copy number analysis including in approximately 900 genes implicated in carcinogenesis. One benign LCT (case #10) showed a copy number deletion in the neurofibromatosis type 2 gene, which was deemed likely pathogenic. The remaining four benign LCTs (cases #6–9, #11) revealed normal gene dosages with no pathogenic CNAs identified. The malignant LCT (case #6) showed a high burden of pathogenic copy number gains and losses as well as loss of heterozygosity in many genes. The sample was deemed most likely to have a complex karyotype with monosomies, trisomies and tetrasomies indicating an overall state of genomic instability. Case #2 (atypical LCT) showed extensive polysomy with increased copy numbers of regions across multiple chromosomes including whole chromosomes. The sample, therefore, revealed a state of genomic instability. Another atypical LCT (case #1) showed increased copy number gains of partial and entire chromosomes, imparting a likely hyperploidy to triploidy tumoural phenotype. Once again, the sample showed a state of genomic instability. The remaining three atypical LCTs (cases #3, #4 and #5) showed normal gene dosage and a lack of pathogenic CNAs identified. Figure 3 demonstrate whole genome profile of tumour DNA analysed by Oncoscan.

## Discussion and Future Directions

In the current study, we analysed 11 cases of testicular LCTs and divided them into ‘benign’, ‘atypical’ and ‘malignant’ categories. Atypical cases were defined by having any of the originally proposed adverse criteria described by Kim *et al*. Malignant cases were defined by the presence of biopsy‐proven metastases. Benign cases lacked any adverse parameters as previously explained. Our cohort consisted of five benign, five atypical and one malignant LCT.

CNV analysis performed on our cohort revealed the presence of several CNAs and subsequent genomic instability in two atypical and one malignant LCT. This is in contrast to benign LCTs which lacked genomic instability. Hypothesis testing performed for this variable did not reveal statistical significance, but this could be due to our small sample size of 11 cases overall. Importantly, only one case in our cohort revealed the presence of recurrence: the malignant LCT (case #6), with biopsy‐proven metastatic recurrence of tumour into the ipsilateral scrotal skin. All other LCTS (benign and malignant) did not reveal any recurrence. Therefore, the presence of genomic instability seemed to not be sufficient for malignant progression and metastatic potential. In addition, LeSS scores were not significantly different between genomic unstable and stable LCTs (*P* = 0.08, data not shown in results). Although two genomically unstable tumours had high LeSS scores (case #2: 5, case #6: 6), the other unstable LCT (case #1) only had an LeSS score of 1. Conversely, case #5 had an LeSS score of 4 but was deemed genomically stable by CNV analysis. Thus, the relationship between genomic instability and adverse histological features remains unclear. Nevertheless, the absence of genomic instability in benign cases coupled with its presence in some atypical and the malignant case, raise the hypothesis of chromosomal instability being an early, but not sufficient, event in the malignant progression of LCTs. The presence of genomic CNAs has been extensively documented in many malignant tumours throughout the body, and the landscape of genomic CNA research, including its therapeutic potential, continues to develop at an astounding pace.^9^, ^10^, ^11^

Future directions for research in this field include delineating specific genes implicated in LCT pathogenesis and how they correlate with tissue expression. This is an evolving area of study, which is adding to our overall understanding of these tumours. For instance, a proportion of malignant LCTs has been shown to harbour mouse double minute 2 (*MDM2*) and cyclin‐dependent kinase 4 (*CDK4*) amplifications, evaluated by fluorescent in situ hybridization and next‐generation sequencing (NGS). These were correlated with strong and diffuse expression of MDM2 and CDK4 by immunohistochemistry (IHC) in 3/11 (27%) of malignant cases, and absence of staining in any of the benign (0/37) cases.^8^ More recently, a study by Rizzo *et al*. using NGS and IHC analysis revealed three distinct groups of aggressive LCTs characterized by FH inactivation, Wnt pathway activation and copy number changes without recurrent mutations, respectively. Beta‐catenin (*CTNNB1*) mutations and adenomatous polyposis coli biallelic inactivations occurred in both aggressive and nonaggressive cases, whereas FH inactivation and copy number changes only occurred in aggressive ones, thus postulating nuclear translocation of β‐catenin and Wnt pathway activation as an early driver event in the progression towards malignant behaviour in LCTs.^12^ Future experiments could look at analysing expression of these genes by IHC. Another study by Kruslin *et al*. using NGS and IHC analysis revealed Telomerase reverse transcriptase mutations in 3/7 successfully analysed cases of metastatic LCTs.^13^ Comparative genome profiling performed by Necchi *et al*. on metastatic testicular SCSTs (10 LCTs, 6 sertoli cell tumours and 3 undifferentiated SCSTs) revealed the absence of microsatellite instability‐high status, and a low tumour mutational burden. The most frequent non‐targetable GA included mutations in *CTNNB1* and cyclin‐dependent kinase inhibitor 2A/B. Targetable Gas were uncommon in all tumours but several showed potential for cell‐cycle inhibitors, hedgehog inhibitors and polymerase inhibitors.^14^

## Conclusion

In summary, our study hypothesis that genomic instability may be an early, but not sufficient, step in the malignant progression of LCTs. Genomic CNV analysis by itself, however, cannot determine which tumours will progress along that spectrum. Metastatic spread continues to be the only definitive criterion for establishing malignancy in such tumours. A key limitation of this study is the very small sample size, with only one malignant case available and no possibility of expanding the cohort or performing additional IHC analyses. Consequently, the conclusion that genomic instability influences the malignant progression of LCTs should be interpreted with caution, as most tumours in this cohort are atypical and exhibit LeSS scores overlapping with those of benign tumours.

## Author contributions

Muhammad F.K. Choudhry: drafted manuscript. Diogo Caires: collection of data and draft manuscript. Yaser Gamallat: data collection. Asli Yilmaz: analysis and interpretation. Fadi Brimo: data acquisition. Bob Argiropoulos: copy number analysis Tarek A. Bismar: conceptual of study, funding acquisition and overall supervision of study.

## Funding information

This study was supported in part by funds from Alberta Precision Laboratory, Resident Research Support Funds.

## Conflict of interests

The authors declare there are no conflicts of interest.

## Patient consent statement

Written informed consent was obtained from all patients for the collection, use and publication of their data in this study.

## Data Availability

The data presented in this study can be produced upon request.

## References

[1] Jou P , Maclennan GT . Leydig cell tumor of the testis. J. Urol. 2009; 181; 2299–2300. 19303105 10.1016/j.juro.2009.02.051

[2] Al‐Obaidy KI , Idrees MT . Testicular tumors: A contemporary update on morphologic, immunohistochemical and molecular features. Adv. Anat. Pathol. 2021; 28; 258–275. 33871428 10.1097/PAP.0000000000000302

[3] Al‐Agha OM , Axiotis CA . An in‐depth look at Leydig cell tumor of the testis. Arch. Pathol. Lab Med. 2007; 131; 311–317. 17284120 10.5858/2007-131-311-AILALC

[4] Bertram KA , Bratloff B , Hodges GF , Davidson H . Treatment of malignant Leydig cell tumor. Cancer 1991; 68; 2324–2329. 1913469 10.1002/1097-0142(19911115)68:10<2324::aid-cncr2820681036>3.0.co;2-k

[5] Kim I , Young RH , Scully RE . Leydig cell tumors of the testis. A clinicopathological analysis of 40 cases and review of the literature. Am. J. Surg. Pathol. 1985; 9; 177–192. 3993830 10.1097/00000478-198503000-00002

[6] Cheville JC , Sebo TJ , Lager DJ , Bostwick DG , Farrow GM . Leydig cell tumor of the testis: A clinicopathologic, DNA content, and MIB‐1 comparison of nonmetastasizing and metastasizing tumors. Am. J. Surg. Pathol. 1998; 22; 1361–1367. 9808128 10.1097/00000478-199811000-00006

[7] Heer R , Jackson MJ , El‐Sherif A , Thomas DJ . Twenty‐nine Leydig cell tumors: Histological features, outcomes and implications for management. Int. J. Urol. 2010; 17; 886–889. 20812939 10.1111/j.1442-2042.2010.02616.x

[8] Colecchia M , Bertolotti A , Paolini B *et al*. The Leydig cell tumour scaled score (LeSS): A method to distinguish benign from malignant cases, with additional correlation with MDM2 and CDK4 amplification. Histopathology 2021; 78; 290–299. 32757426 10.1111/his.14225

[9] Leary RJ , Lin JC , Cummins J *et al*. Integrated analysis of homozygous deletions, focal amplifications, and sequence alterations in breast and colorectal cancers. Proc. Natl. Acad. Sci. USA 2008; 105; 16224–16229. 18852474 10.1073/pnas.0808041105 PMC2571022

[10] Erickson A , He M , Berglund E *et al*. Spatially resolved clonal copy number alterations in benign and malignant tissue. Nature 2022; 608; 360–367. 35948708 10.1038/s41586-022-05023-2 PMC9365699

[11] Grist E , Friedrich S , Brawley C *et al*. Accumulation of copy number alterations and clinical progression across advanced prostate cancer. Genome Med. 2022; 14; 102. 36059000 10.1186/s13073-022-01080-4 PMC9442998

[12] Rizzo NM , Sholl LM , Idrees MT *et al*. Comparative molecular analysis of testicular Leydig cell tumors demonstrates distinct subsets of neoplasms with aggressive histopathologic features. Mod. Pathol. 2021; 34; 1935–1946. 34103665 10.1038/s41379-021-00845-3

[13] Kruslin B , Gatalica Z , Hes O *et al*. TERT gene fusions characterize a subset of metastatic Leydig cell tumors. Clin. Genitourin. Cancer 2021; 19; 333–338. 33741265 10.1016/j.clgc.2021.02.002 PMC9907364

[14] Necchi A , Bratslavsky G , Shapiro O *et al*. Genomic features of metastatic testicular sex cord stromal tumors. Eur. Urol. Focus 2019; 5; 748–755. 31147264 10.1016/j.euf.2019.05.012

